# Retraction Note: Inhibition of microRNA-214 promotes epithelial–mesenchymal transition process and induces interstitial cystitis in postmenopausal women by upregulating Mfn2

**DOI:** 10.1038/s12276-022-00787-z

**Published:** 2022-06-14

**Authors:** Jian-Wei Lv, Wei Wen, Chen Jiang, Qi-Bo Fu, Yin-Jun Gu, Ting-Ting Lv, Zhen-Dong Li, Wei Xue

**Affiliations:** 1 Department of Urology, South Campus, Ren Ji Hospital, School of Medicine, Shanghai Jiaotong University, Shanghai, China; 2 Department of Urology, Shanghai General Hospital, School of Medicine, Shanghai Jiao Tong University, Shanghai, China; 3 Department of Urology, Ren Ji Hospital, School of Medicine, Shanghai Jiaotong University, Shanghai, China

Retraction to: *Experimental and Molecular Medicine* 10.1038/emm.2017.98, published online 21 July 2017

The authors have retracted this article because of issues with two of the figures. Specifically:

- Figures 3a and 3b overlap with Figures 2b and 2c in Ref. ^1^
- Figure 3b overlaps with Figure 2C in Ref. ^2^

All authors agree with this retraction.
